# Successful Neoadjuvant Chemotherapy for Small-Cell Neuroendocrine Carcinoma of the Pancreas: A Case Report

**DOI:** 10.3389/fonc.2021.719422

**Published:** 2021-09-10

**Authors:** Keyu Li, Jialong Yuan, Yichen Li, Hao Zhang, Xubao Liu, Nengwen Ke

**Affiliations:** ^1^Department of Hepato-Bilio-Pancreatic Surgery, West China Hospital, Sichuan University, Chengdu, China; ^2^School of Basic Medical Sciences, Capital Medical University, Beijing, China

**Keywords:** neuroendocrine carcinoma, neoadjuvant treatment, chemotherapy, pancreatic cancer, surgery

## Abstract

Neoadjuvant therapy for pancreatic neuroendocrine tumors may potentially aid downstaging, increase the possibility of radical surgery. We herein report a case of a 63-year-old man who had been diagnosed with locally advanced small-cell neuroendocrine carcinomas of the pancreas according to the diagnostic biopsy. The patient received 6 courses of etoposide and cisplatin as neoadjuvant therapy in an attempt to stop tumor progression, which promoted obvious tumor shrinkage without adverse effects and allowed subsequent Appleby procedure, the distal pancreatectomy with celiac artery resection. The patient showed no recurrence in the follow-up of a contrast-enhanced computed tomographic scan, which is 8 months after surgery. To the best of our knowledge, this is a rare case to report etoposide and cisplatin administration before surgery for unresectable pancreatic neuroendocrine carcinoma promoted a pathological partial response and finally achieved a radical surgery, providing a novel therapeutic option for patients with locally advanced pancreatic neuroendocrine carcinoma.

## Introduction

Patients with poorly differentiated pancreatic neuroendocrine carcinomas (pNECs) typically present with an aggressive course, frequent metastases, and poor prognosis (1). Given the aggressive biologic behavior does not appear to be impacted by surgical resection, radical surgery is not recommended in the clinical guidelines (2). However, research from the SEER database for pNECs demonstrated that removal of the primary tumor significantly correlated with improved overall survival (3). For locally advanced pancreatic neuroendocrine tumors (pNETs), a recent study reported that patients who underwent surgical resection had excellent disease-free and overall survival (4). Neoadjuvant therapy represents an option for downstaging of selected patients with advanced and metastatic pNETs or pNECs, thus rendering curative resection feasible (5, 6). Several systemic agents have been introduced for neoadjuvant treatment in pNETs, which include multi-agent chemotherapy, targeted therapy, somatostatin analogues, and peptide receptor radionuclide therapy (7–10). A recent encouraging result from the E2211 clinical trial confirmed the value of capecitabine and temozolomide for PFS benefit in patients with advanced pNETs (11). However, few studies have explored neoadjuvant treatments for pNEC. Here, we report a case of small-cell pNEC using etoposide and cisplatin as neoadjuvant intervention and subsequent receiving safe distal pancreatectomy with celiac artery resection.

## Case Description

**Figure 1:**
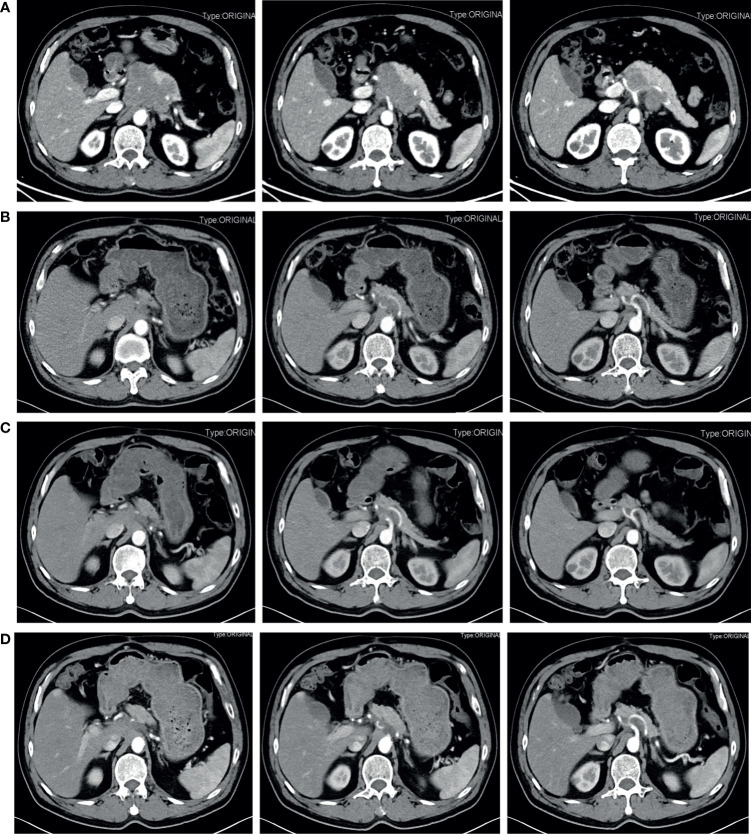
Contrast-enhanced abdominal CT during treatment before the radical surgery. **(A)** Pretreatment. A retroperitoneal neoplastic lesion measuring about 6.5 cm x 8.3 cm was observed, which was located on the head and neck of the pancreas and manifested a moderate enhancement. The left gastric artery and splenic artery were covered in the tumor, and the common hepatic artery and the celiac trunk were got invaded. **(B)** 3 months after four courses of etoposide plus cisplatin neoadjuvant chemotherapy. The tumor size was significantly reduced and limited to 2.7 cm x 2.3 cm. **(C)** 5 months after four courses of etoposide plus cisplatin neoadjuvant chemotherapy. The tumor size was limited to 2.7 cm x 2.3 cm. **(D)** After six courses of neoadjuvant chemotherapy. The tumor size reached 3.0 cm x 2.2 cm.

The patient is a 63-year-old male first seen in our hospital due to a complaint of intermittent mid-upper abdominal pain. He had a free previous medical history. The physical examination was normal. An enhanced abdominal computed tomography (CT) scan was requested, which showed a retroperitoneal neoplastic lesion measuring about 6.5 cm x 8.3 cm. The lesion was located on the head and neck of the pancreas and manifested a moderate enhancement. The left gastric artery and splenic artery were covered in the tumor, and the common hepatic artery and the celiac trunk were got invaded (**Figure 1A**). Serological carbohydrate antigen 19-9 was normal (23.65 U/ml). Based on the previous clinical evidence, the patient was suspected as a pNET. Considering that pNET is not as malignant as pancreatic ductal adenocarcinoma, and preoperative CT suggested that there might be an approach for distal pancreatectomy with celiac artery resection to achieve radical resection (R0), an exploratory laparotomy was performed. However, the surgery found that tumor invasion was deeper than preoperative evaluation, and the liver arterial supply might not be guaranteed after resection. The patient finally only received a biopsy during the exploratory laparotomy. Postoperative histopathological examination confirmed the diagnosis with small-cell neuroendocrine carcinomas of the pancreas. Further immunohistochemistry showed that the tumor epithelial cells were positive for chromogranin A, synaptophysin, and Ki-67 (Approximately 80%). Commercial tumor Next-Generation Sequencing analysis indicated a mutational load of 8.06, with microsatellite stability, wild type BRAF, wild type BRCA, wild type KRAS, wild type NRAS, and wild type PIK3CA. Considering the poor prognosis of pNEC, the multidisciplinary team discussion suggested neoadjuvant therapy. As the 68Ga-DOTATOC PET/CT scan had not been introduced in the institution, a 18F-FDG PET/CT scan was requested before the initiation of neoadjuvant therapy, which confirmed that the pNEC was limited to the retroperitoneum. The patient then began 4 courses of etoposide (100 mg/m2 d1-3, Q21d) and cisplatin (50 mg/m2 d1, Q21d) neoadjuvant therapy, and finally achieved a partial response (PR) according to the Response Evaluation Criteria in Solid Tumors (RECIST). 3 months after the fourth neoadjuvant chemotherapy, an enhanced abdominal CT scan was requested and showed a significant decrease of the tumor size, which was limited to 2.7 cm x 2.3 cm (**Figure 1B**). 2 months later, the fifth and sixth course of the neoadjuvant chemotherapy was initiated as the reduction of the tumor size stopped (**Figure 1C**). After a total of six courses of neoadjuvant chemotherapy without any adverse events, he was admitted to our department to evaluate whether a complete resection should be considered.

**Figure 2:**
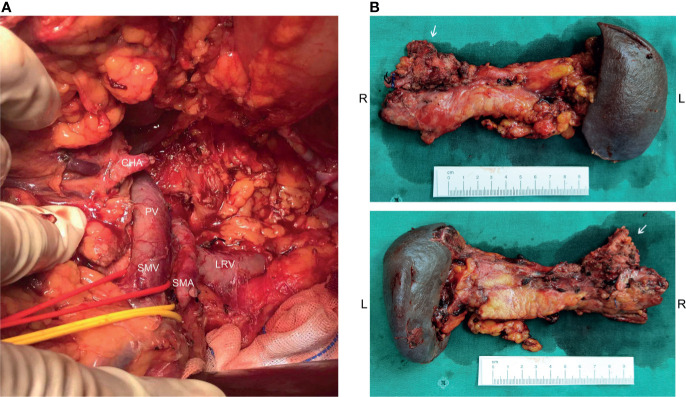
Operative findings and surgical specimen. **(A)** From the operative exploration, the tumor quality was tough, and about 3.0 cm x 2.0 cm x 4.0 cm in diameter, infiltrating the neck and body of the pancreas, invading the lesser curvature, left adrenal gland, and the left diaphragm. The tumor also invaded the celiac trunk, the common hepatic artery, the left gastric artery, the splenic artery, the splenic vein, and its branches. **(B)** An Appleby procedure was performed to completely remove the tumor, tumor invaded vessels, the distal pancreas, and the spleen.

Before the re-evaluation multidisciplinary consultation, an abdominal CT scan revealed a 3.0 cm x 2.2 cm lesion at the original tumor site. Following contrast media administration, the retroperitoneal lesion showed slight to moderate enhancement, with an unclear boundary to the neck and the body of the pancreas and the diaphragm. The tumor had contacted the celiac trunk and its branches over 180° (**Figure 1D**). Serological carbohydrate antigen 19-9 remained normal. The oncologist suggested that radical surgery should be evaluated by surgeons at this moment, given the tumor did not show sufficient shrinkage to qualify for PR nor sufficient increase to qualify for progressive disease (PD) after the fifth and sixth neoadjuvant treatment. Senior surgeons discussed the possibility of radical resection of the primary tumor and risks of vessel replacement as the observed common hepatic artery stenosis and left gastric artery stenosis, though their shape was normal. Finally, given the significant shrinkage of the primary tumor and the absence of adverse events, the patient was subjected to programmed exploratory laparotomy. The tumor was tough, and about 3.0 cm x 2.0 cm x 4.0 cm in diameter, which infiltrated the neck and body of the pancreas, invaded the lesser curvature, left adrenal gland, and the left diaphragm. The tumor also invaded the celiac trunk, the common hepatic artery, the left gastric artery, the splenic artery, the splenic vein, and its branches (**Figure 2A**). Finally, an Appleby procedure, the distal pancreatectomy with celiac artery resection, was performed to completely remove the tumor and tumor invaded arteries and veins (**Figure 2B**).

**Figure 3:**
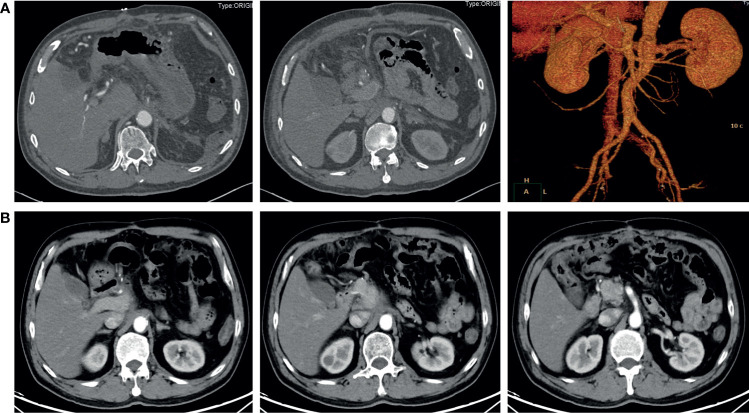
Contrast-enhanced abdominal CT after surgery. **(A)** 6 days after the radical surgery. The distal pancreas, spleen, and celiac trunk were absent. **(B)** 8 months after the radical surgery. No signs of recurrence or metastasis were found.

Although the tumor had invaded the celiac trunk, all dissected lymph nodes were free of involvement. Instead of cancer cells, only necrotic and fibrotic tissues were detected in the postoperative pathological examination. The postoperative CT scan demonstrated an uneventful perioperative course, and the patient was discharged 7 days after the surgery (**Figure 3A**). The latest follow-up was 8 months after surgery, and no signs of recurrence or metastasis were found in the CT scan (**Figure 3B**).

## Discussion

Neuroendocrine neoplasms (NEN) originate from neuroendocrine cells and may produce a variety of hormones with neuroendocrine phenotypes. According to the 2019 World Health Organization (WHO) Classification of Digestive System Tumors, the proliferation activity of gastroenteropancreatic NETs cells is evaluated by the number of mitotic counts or Ki-67 proliferation index. Pancreatic neuroendocrine neoplasms can be classified into well-differentiated pNETs and poorly differentiated pNECs (12). The annual incidence of pNETs has increased in the United States over the last 2 decades from 0.3 to 1.0 cases per 100,000 per year (13). Besides, more than a third of patients have metastatic disease, and an additional 20% have locally advanced disease when diagnosed with NETs. The incidence in China is also increased yearly, and the majority of patients have non-functional tumors (14). In this present case, the pathological examination revealed that the tumor is a small-cell pNEC, which usually with an aggressive course, and a poor prognosis.

Preoperative evaluation is very important as the majority of patients with pancreatic and gastrointestinal neuroendocrine tumors benefit from surgical management. Imaging like contrast-enhanced CT scan or magnetic resonance imaging scan is generally utilized for determining the invasion to the adjacent organs, whether the tumor has metastasized to the lymph node causing its enlargement, and even the distant metastases. The NEC presenting in this case report has contacted the celiac trunk and common hepatic artery for over 180° and compressed the splenic artery and the left gastric artery with focal vessels narrowing. According to the 2017 ENETS guidelines, this NEC should be categorized as a locally advanced tumor, which was recommended to receive systemic therapies based on their performance status (15). In the latest 2020 North American Neuroendocrine Tumor Treatment Guidelines, EP regimen, the combination of cisplatin or carboplatin and etoposide, is the first-line option for NEC (16, 17). Irinotecan as an alternative to etoposide is also acceptable (18). Somatostatin analogue like octreotate, and targeted therapies, such as everolimus or sunitinib, are not thought to benefit patients with NECs (9, 19). Literature has reported that everolimus and octreotate could be useful for neoadjuvant therapy in cases of locally advanced pancreatic neuroendocrine tumors to achieve successful conversion to surgical resection (8, 20). However, results of other studies remain ambiguous (7).

The main purpose of neoadjuvant chemotherapy is to shrink tumors and kill invisible metastatic cells as soon as possible to facilitate subsequent treatment, which refers to systemic chemotherapy done before local treatments like surgery or radiotherapy. Increasing evidence demonstrates the application of neoadjuvant therapies in advanced gastroenteropancreatic NETs aiming at tumor downsizing, thus rendering curative resection feasible (5, 6). We decided to follow the EP regimen as a neoadjuvant treatment for this patient in an attempt to stop tumor progression. The patient was treated by a drug regimen (etoposide 100mg d1-5 + cisplatin 50mg d1-3, q3w) for a total of 6 cycles, and a CT scan was reviewed after the fourth cycle of medication. The imaging examination showed that the lesion was significantly reduced and the patient was sensitive to chemotherapy. After evaluation through multidisciplinary consultation, a PR was achieved and radical surgery should be considered. The patient then received distal pancreatic surgery to completely remove the primary tumor. We observed an obvious reduction of the tumor size in the operation, and the following postoperative pathological examination confirmed that all tumor cells were disappeared. Follow-up by CT scans did not show any signs of recurrence or metastasis 8 months after surgery.

The present case is an encouraging successful transition for unresectable pNEC through neoadjuvant therapy, which represents an option for downstaging of selected patients with advanced and metastatic pNETs or pNECs. Suitable and sensitive neoadjuvant therapies may achieve better tumor degression and increase the possibility of radical surgery. We hope more relevant clinical cases will be reported in the future to provide more evidence for the neoadjuvant treatment of pNEC.

## Data Availability Statement

The original contributions presented in the study are included in the article/supplementary material. Further inquiries can be directed to the corresponding author.

## Ethics Statement

Ethical review and approval was not required for the study on human participants in accordance with the local legislation and institutional requirements. The patients/participants provided their written informed consent to participate in this study. Written informed consent was obtained from the individual(s) for the publication of any potentially identifiable images or data included in this article.

## Author Contributions

NK contributed to study concept and design. KL and JY contributed to investigation and writing the original draft. YL contributed to data collection. HZ, XL, and NK revised the paper. All authors contributed to the article and approved the submitted version.

## Funding

This research was supported by Sichuan Province Science and Technology Planning Project (2020YFS0262), West China Hospital Clinical Research Incubation Project (21HXFH058), and the 1·3·5 Project for Disciplines of Excellence–Clinical Research Incubation Project (ZY2017302), West China Hospital, Sichuan University.

## Conflict of Interest

The authors declare that the research was conducted in the absence of any commercial or financial relationships that could be construed as a potential conflict of interest.

## Publisher’s Note

All claims expressed in this article are solely those of the authors and do not necessarily represent those of their affiliated organizations, or those of the publisher, the editors and the reviewers. Any product that may be evaluated in this article, or claim that may be made by its manufacturer, is not guaranteed or endorsed by the publisher.
